# Supplementary material for: DeepMethylation: a deep learning based framework with GloVe and Transformer encoder for DNA methylation prediction
Source: PeerJ. 2023 Sep 25;11:e16125. doi: 10.7717/peerj.16125 (PMC10538282; doi:10.7717/peerj.16125)
Supplement: Supplemental Information 1 [file peerj-11-16125-s001.zip › git/RGloVe/text8.zip]

中国矿业大学校园网认证系统


语言 / Language
中文 (简体)
English

### 校园网登录

*person*
账号

*lock*
密码

记住密码

找回密码

充值缴费

登录
自助服务

[](./static/themes/pro/video/video.mp4)

校园网故障报修电话: 400-008-9233，83592139

校园网服务监督电话: 83592122

校园网账户服务电话: 83592138
